# A128 OPTIMIZED COMPUTER ASSISTED TECHNIQUE FOR INCREASING ADENOMA DETECTION DURING COLONOSCOPY: A RANDOMIZED CONTROLLED TRIAL

**DOI:** 10.1093/jcag/gwad061.128

**Published:** 2024-02-14

**Authors:** R Djinbachian, M Taghiakbari, A Barkun, E Medawar, B Panzini, S Sidani, J Liu, D von Renteln

**Affiliations:** CRCHUM, Montréal, QC, Canada; CRCHUM, Montréal, QC, Canada; MUHC, Montreal, QC, Canada; CRCHUM, Montréal, QC, Canada; CRCHUM, Montréal, QC, Canada; CRCHUM, Montréal, QC, Canada; CRCHUM, Montréal, QC, Canada; CRCHUM, Montréal, QC, Canada

## Abstract

**Background:**

Efforts to improve colonoscopy have recently focused on improving adenoma detection interventions such as artificial intelligence (CADe) that have demonstrated improvements in Adenoma Detection Rates (ADR). Although studies have focused on implementation of one intervention at a time.

**Aims:**

We evaluated an optimized computer assisted technique (CADopt) versus standard colonoscopy to improve ADR during colonosocopy.

**Methods:**

A prospective randomized controlled trial was conducted enrolling adults (45–80 years) undergoing elective colonoscopy. Participants were randomized (1:1) to the intervention group (CADopt, and control group. In the CADopt group, endoscopists used a computer aided polyp detection combined with linked colour imaging, water exchange colonoscopy, and cecal retroflexion. In the control group, standard colonoscopy was performed. Primary outcome was Adenoma Detection Rate (ADR) in the intervention and control groups. Secondary outcomes included advanced ADR (AADR) and sessile serrated lesion detection rates (SDR).

**Results:**

A total of 467 patients were recruited and randomized (CADopt group 229 patients, 50.2% female vs 238 patients, 48.3% female in the control group). ADR was 49.3% (95%CI 42.7-56.0) for the CADopt group vs 38.2% (95%CI 32.0-44.7) for the control group (p=0.016). AADR and SDR were 13.1% (95%CI 9.0-18.2) and 6.6% (95%CI 3.7-10.6) for the CADopt group versus 8.0% (95%CI 4.9-12.2), and 7.1% (95%CI 4.2-11.1) for the control group, respectively.

**Conclusions:**

Using an optimized computer assisted technique led to significant improvements in ADR and a trend towards AADR improvements.

**Funding Agencies:**

Fujifilm

